# Immune-related adverse events of immune checkpoint inhibitors: a review

**DOI:** 10.3389/fimmu.2023.1167975

**Published:** 2023-05-25

**Authors:** Qinan Yin, Liuyun Wu, Lizhu Han, Xingyue Zheng, Rongsheng Tong, Lian Li, Lan Bai, Yuan Bian

**Affiliations:** ^1^ Department of Pharmacy, Sichuan Academy of Medical Sciences & Sichuan Provincial People’s Hospital, School of Medicine, University of Electronic Science and Technology of China, Chengdu, China; ^2^ Personalized Drug Therapy Key Laboratory of Sichuan Province, School of Medicine, University of Electronic Science and Technology of China, Chengdu, China

**Keywords:** immune checkpoint inhibitors, immune-related adverse events, epidemiology, mechanism, management

## Abstract

Since the first Immune Checkpoint Inhibitor was developed, tumor immunotherapy has entered a new era, and the response rate and survival rate of many cancers have also been improved. Despite the success of immune checkpoint inhibitors, resistance limits the number of patients who can achieve a lasting response, and immune-related adverse events complicate treatment. The mechanism of immune-related adverse events (irAEs) is unclear. We summarize and discuss the mechanisms of action of immune checkpoint inhibitors, the different types of immune-related adverse events and their possible mechanisms, and describe possible strategies and targets for prevention and therapeutic interventions to mitigate them.

## 1 Introduction

At the end of the 19th century, the fields of immunology and oncology were linked when surgeon William Coley reported that injecting inactivated bacteria into sarcomas could bring about tumor shrinkage (1). In recent years, great breakthroughs have been made in tumor immunotherapy, which have significantly improved the survival rate of cancer patients. To date, there have been various types of immunotherapy drugs, including tumor vaccines, cellular immunotherapy, immunomodulatory drugs targeting T cells, and immune checkpoint inhibitors (ICIs). With the development of a variety of new high-tech technologies, the means of tumor immunotherapy are also constantly enriched. However, in clinical practice, chemotherapy and radiotherapy remain the mainstay of treatment for most cancer types, and ICIs are still the first-line treatment for a variety of solid and liquid tumors (2). ICIs are a modality of antitumor drugs. However, with the increased use of ICIs, the number of immune-related adverse events (irAEs) increases. Different from the typical adverse reactions of radiotherapy and chemotherapy, patients have varying reactions to immunotherapy. IrAEs may occur with significant changes in tumor size and appearance after treatment for several months and even the whole course of ICIs (3). irAEs are unique, unlike conventional cancer therapies. They often have a delayed onset and prolonged course. IrAEs can involve any organ or system, usually with low impact, and are treatable and reversible, but some adverse effects can be serious and lead to permanent illness (4).

This review, using keywords of immune checkpoint inhibitors and immune-related adverse events, was performed with databases such as Web of Science, CNKI, and MEDLINE. Most of the articles included were published within the last 5 years. ICI treatment offers cancer patients considerable promise of survival, whereas awareness of irAEs is needed by means of close collaboration across multiple disciplines, as ICIs are still in their infancy in being approved for oncology treatment. Previous reviews or literature have mostly studied irAEs in specific organs or systems, and there is a lack of systematic summaries of the mechanism and management of irAEs. This review introduces the mechanism of ICIs, related adverse immune reactions, and related management. We sincerely expect that medical practitioners will be assisted in diagnosing, preventing, and treating irAEs through this article and that the clinical application of ICIs will be further considered.

## 2 Mechanisms and representative drugs

**Figure 1:**
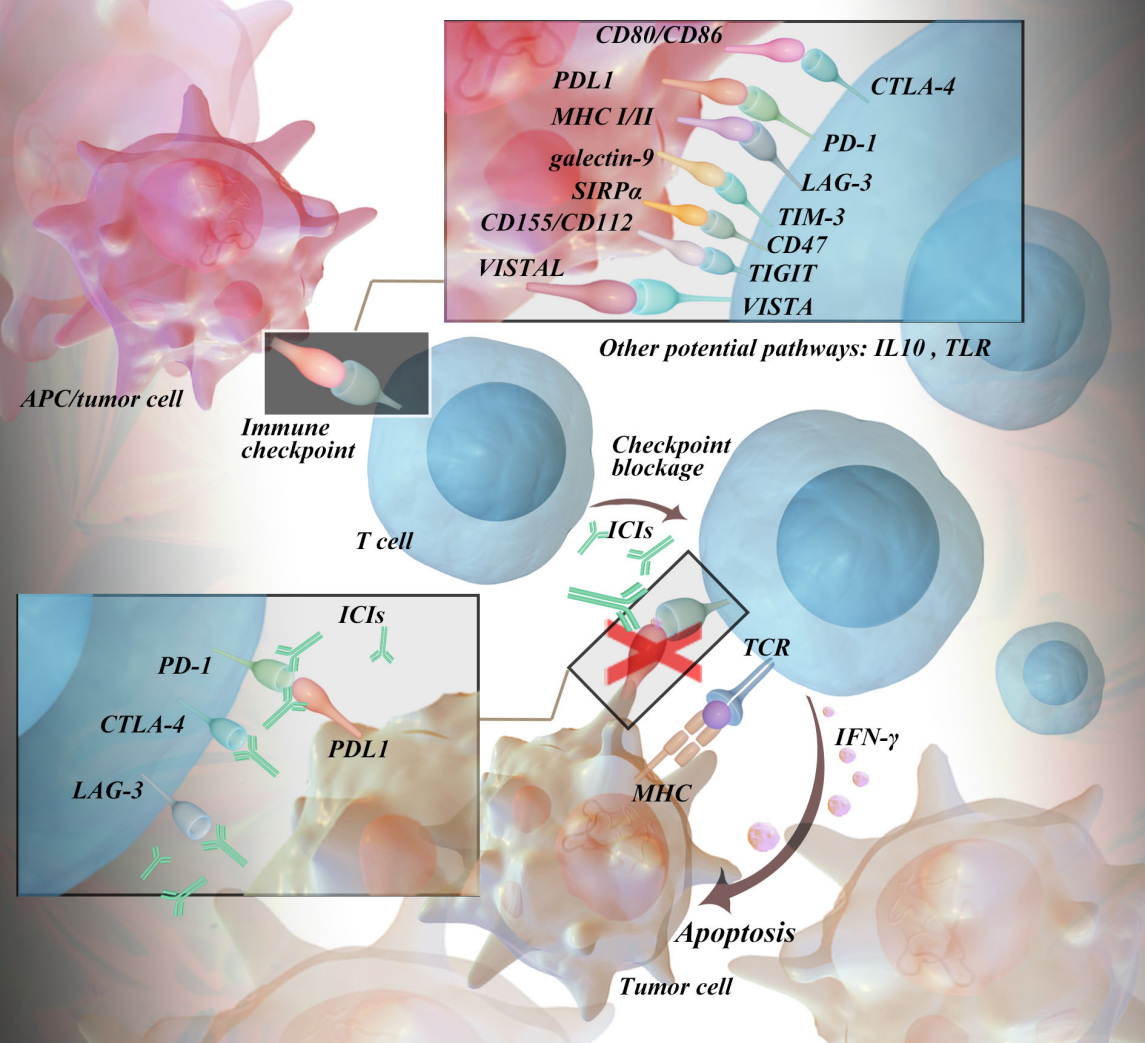
The Action Mechanisms of Immune Checkpoints.

In most cases, the immune system eliminates cancer cells in early therapy. In addition, cancer cells can develop various mechanisms to evade the immune system, which in turn leads to advanced disease (5). Immune checkpoints are one of the mechanisms by which cancer cells disguise themselves in the body. It is a negative regulator of the immune system, mediating self-tolerance, preventing autoimmunity, and protecting tissues from immune attack (6). This mechanism is often exploited by tumor cells to evade immune surveillance (7). It can also be understood as a restrictive and inhibitory pathway in the immune system, which can downregulate the function of the immune system, promote the function of regulatory immune cells, and produce immunosuppressive cytokines and chemokines. T lymphocytes (also called T cells) are the core of cell-mediated immunity. Activated T cells can secrete a large number of cytokines to upregulate immune checkpoints (8, 9). Tumor cells inhibit the activation of T cells by activating certain immune checkpoint proteins, which eventually leads to enhanced immune resistance of tumor cells (10). To date, the identified immune checkpoints mainly include programmed death 1 (PD-1) and its ligand 1 (programmed death-ligand 1, PD-L1), cytotoxic T lymphocyte-associated antigen 4 (CTLA-4), and lymphocyte-activation gene 3 (LAG-3). Other checkpoints include T-cell immunoglobulin and mucin domain-containing protein 3 (TIM-3), CD47, T-cell immunoglobulin and ITIM domain protein (TIGIT), and V-domain Ig suppressor of T-cell activation (VISTA) (11). PD-1, or CD279, belongs to the CD28 family and is a coinhibitory transmembrane protein expressed on antigen-stimulated T and B lymphocytes, natural killer cells (NK), and myelosuppressive dendritic cells (MDSCs). After binding to the corresponding ligands, they can reduce the response of T cells to T-cell receptor (TCR) stimulation signals and regulate the intensity of the immune response (12, 13). Current research on PD-1 ligands mainly focus on PD-L1 since the role of PD-L2 in tumor immunosuppression is controversial. PD-L1 can be expressed by tumor cells, epithelial cells, dendritic cells, macrophages, fibroblasts, and depleted T cells, and its expression intensity is influenced by cytokines (such as IFN-γ) and carcinogenic factors. As PD-L1 binds to PD-1, the PISK-AKT and Ras-Raf-MEK-ERK signaling pathways are suppressed, thus inhibiting the proliferation and differentiation of effector T cells (14–16). CTLA-4 is a type I transmembrane glycoprotein of the immunoglobulin superfamily that is highly expressed in tumor tissues, commonly present in the cytoplasm of CD4+ and CD8+ T cells, and considered a negative regulator of antitumor immunity. It can be induced on the cell surface, binds to CD80 (B7-1) and CD86 (B7-2) on the surface of antigen presenting cells (APCs), and has a higher affinity than the costimulatory molecule CD28 of T cells, thereby inhibiting cytotoxic T-cell activity and enhancing regulatory T-cell (Treg) immunosuppressive activity, causing the immune evasion of tumor cells (17, 18). LAG-3 is a transmembrane protein that can be constitutively expressed or induced on a variety of immune cells, such as CD4/CD8+ T cells, natural killer (NK) cells, invariant NK T cells, plasma-like DCs (pDCs) and B cells, and is often co-expressed with other checkpoints, such as PD-1 and CTLA-4. Its extracellular domain, consisting of 4 immunoglobulins, has 20% amino acid identity with that of CD4, and only the genomic regions encoding intracellular regions between them differ, resulting in different functions (19). The binding of LAG-3 to its ligand can hinder antitumor cellular immunity, leading to tumor immune evasion (20). MHC II molecules are considered the canonical ligand of LAG-3 (20–22), and other ligands have been discovered later, such as fibrinogen-like protein 1 (23), liver sinusoidal endothelial cell lectin (24), galectin-3 (25), and α-synuclein (26). LAG-3 expression was positively correlated with the expression of almost all MHC-related genes in various cancers. Currently, other ICIs, including TIM-3, CD47, TIGIT, and VISTA, are being extensively studied and developed in clinical trials. Binding with its typical ligand galectin-9 (Gal-9), TIM-3 can mediate the dysfunction and exhaustion of TIM-3+ T cells. Preclinical studies have shown that anti-TIM-3 therapy exhibits antitumor effects in various mouse models (27). CD47 is overexpressed in multiple types of solid tumors and hematologic malignancies, evading surveillance by macrophages through interaction with its ligand signal regulatory protein α (SIRPα) and inhibiting macrophage-mediated clearance of tumor cells. In preclinical studies, CD47 antibodies have demonstrated antitumor activity in a variety of malignancies. TIGHT is primarily expressed in T cells and NK cells and functions by inhibiting the antitumor activity of T cells and NK cells through its binding with CD155. VISTA shares homology with PD-L1 and PD-L2, and is highly expressed in myeloid-derived suppressor cells and immune cells. When bound to V-set and immunoglobulin domain containing 3 (VSIG3) and P-selectin glycoprotein ligand 1 (PSGL-1), VISTA exerts inhibitory effects on T cells. The mechanism is detailed in **Figure 1**.

Immune checkpoint blockade is designed to interfere with inhibitory pathways that naturally constrain T-cell reactivity, therefore releasing inherent limits on the activation and maintenance of T-cell effector function (28). With the in-depth study of the mechanism of immune checkpoints, ICIs such as CTLA-4 and PD-1/PD-L1 have shown good antitumor activity in malignant tumors such as urothelial carcinoma, renal cell carcinoma, melanoma, non-small cell lung cancer, colorectal cancer, and Hodgkin lymphoma and have been widely used in clinical practice (29). As of November 2022, 10 ICIs by the United States Food and Drug Administration (FDA), 16 ICIs by the National Medical Products Administration (NMPA) of China and 11 ICIs by the European Medicines Agency (EMA) have been approved for the treatment of different malignancies. The commonly used immune checkpoint inhibitors worldwide include main three categories: 1) anti-PD-1/PD-L1 monoclonal antibodies such as nivolumab, pembrolizumab, atezolizumab, durvalumab, avelumab, and cemiplimab; 2) anti-CTLA-4 monoclonal antibodies, such as ipilimumab and tremelimumab; and 3) anti-PD-1 and CTLA-4 combination inhibitors, such as nivolumab in combination with ipilimumab. These drugs are widely used in the treatment of various malignancies and have become a hot research topic in the field of cancer immunotherapy. The approval status of commonly used ICI is shown in **Table 1** (including FDA, NMPA, EMA, etc.).

**Table 1:** The approval status of commonly used ICI agents in clinical practice.

Targets	Drugs	Approved by	Indications
CTLA-4	Ipilimumab	FDA, EMA, HC, NMPA	Advanced MM, advanced CRC, NSCLC, HL
PD-1	Nivolumab	FDA, EMA, HC, MHLW, NMPA	HNSCC, NSCLC, GC, ESCC, CRC, HCC, RCC, HL, MM, skin cancer
	Pembrolizumab	FDA, EMA, HC, NMPA, NMPA	HNSCC, NSCLC, GC, ESCC, CRC, HCC, RCC, HL, MM, skin cancer, TNBC
	Cemiplimab	FDA, EMA, HC	skin cancer, NSCLC
	Toripalimab	NMPA, EMA	skin cancer, HNSCC, BRCA
	Sintilimab	NMPA, EMA, FDA	NSCLC, HCC, HL
	Camrelizumab	NMPA, FDA	NSCLC, HCC, HL, ESCC, HNSCC
	Tislelizumab	NMPA	NSCLC, HL, BRCA
	Penpulimab	NMPA	HCC, GC, NSCLC, NPC, HL
	Zimberelimab	NMPA	HL
	Serplulimab	NMPA	GC, CRC, NSCLC
	Pucotenlimab	NMPA	CRC, MM
	Dostarlimab	FDA, EMA, HC	UCEC
PD-L1	Durvalumab	FDA, EMA, HC, NMPA	NSCLC, SCLC, BRCA
	Atezolizumab	FDA, EMA, HC, NMPA	skin cancer, NSCLC, SCLC, BRCA, HCC, TNBC
	Envafolimab	NMPA	CRC
	Sugemalimab	NMPA	NSCLC
	Avelumab	FDA, EMA, HC	skin cancer, RCC, BRCA
LAG-3	Relatlimab	FDA, EMA	Metastatic MM

EMA, European Medicines Agency;FDA, United States Food and Drug Administration; HC, Health Canada; MHLW, Ministry of Health, Labour and Welfare of Japan; NMPA, National Medical Products Administration of China; BCC, basal cell carcinoma; BRCA, bladder urothelial carcinoma; CC, cervical cancer; cHL, classical Hodgkin’s lymphoma; CRC, colorectal cancer; CSCC, cutaneous squamous cell carcinoma; dMMR, mismatch repair deficiency; EC, endometrial carcinoma; ESCC, esophageal squamous cell carcinoma; GC, gastric cancer; HCC, hepatocellular carcinoma; HL, Hodgkin’s lymphoma; HNSCC, head and neck squamous cell carcinoma; MCC, Merkel cell carcinoma; MM, malignant melanoma; MSI-H, high microsatellite instability; MPM, malignant pleural mesothelioma; NSCLC, non-small cell lung cancer; NPC, nasopharyngeal carcinoma; PMBCL, primary mediastinal large B cell lymphoma; PMF, primary myelofibrosis; RCC, renal cell carcinoma; SCLC, small cell lung cancer; TMB-H, high tumor mutation burden; TNBC, triple-negative breast cancer; UC, urothelial carcinoma; UCEC, uterine corpus endometrial carcinoma.

## 3 Epidemiology of adverse reactions to ICIs

ICIs break the immune balance of the body and reduce T-cell tolerance, leading to the production of a series of irAEs (30). To grade the severity of irAEs, we took the Common Terminology Criteria for Adverse Events (CTCAE) commonly by the National Cancer Institute, which classifies toxicities into 5 ascending symptoms: asymptomatic/mild (grade 1), moderate (grade 2), severe (grade 3), life-threatening (grade 4), and death (grade 5) (31). IrAEs are common and have been reported to occur in 90% of patients treated with anti-CTLA-4 and 70% of patients treated with anti-PD-1/PD-L1 (32). The incidence of single-agent irAEs ranges from 15% to 90% (33). In a systematic review of 50 trials, the incidence of grade 3/4 AE was 21% (range 0%–66%) (34). Anti-CTLA-4 therapy was associated with a higher incidence of adverse effects than anti-PD-1 and PD-L1 therapy, and combination therapy was associated with a higher incidence of adverse effects than monotherapy (35). In addition, a meta-analysis by Mi et al. further illuminated that the incidence of irAEs was higher with APLC therapy (PD-L1 inhibitors plus CTLA-4 inhibitors) than with APC therapy (PD-1 inhibitors plus CTLA-4 inhibitors), and the 26 included studies reported more than 50 kinds of treatment-related adverse events, with a rate of 65.7% in all grades (36). The systematic review by Anne et al. included a total of 1265 patients from 22 clinical trials of CTLA-4 inhibitors, with a dose-dependent prevalence of 72% and 24% of overall and advanced irAEs, respectively, and 0.86% of patients dying due to irAEs (37). A recent systematic review of 125 clinical trials involving 20128 patients showed an overall incidence of 66.0% of irAEs of all grades and 14.0% of grade 3 or above following treatment with PD-1 or PD-L1 inhibitors. ICI-related irAEs are organ specific, with skin-related irAEs being the most common (especially mild itching or rash), followed by gastrointestinal toxicity, often manifested as diarrhea and colitis (38, 39); the third most common is endocrine irAEs (40), including thyroid dysfunction (hypothyroidism and hyperthyroidism), pituitary inflammation, and adrenal insufficiency; musculoskeletal toxicity (such as mild joint pain or muscle pain) and ocular toxicity (such as mild dry eye syndrome and uveitis) are also frequently reported (32). Pneumonia, myocarditis, neurotoxicity, myositis, nephritis, and hematological toxicity are not very common, but the potential seriousness is worth noting. The majority of patients who experience these irAEs have a mortality rate between 10% and 17%, and the mortality rate for myocarditis is extremely high at 39.7%. Neurotoxicity is usually more serious, with encephalitis and severe myasthenia gravis being the most common fatal diseases (41). Overall mean adverse event rates were similar for different cancer types but different among drugs (42). CTLA-4 inhibitors often cause colitis, hypophysitis, and rash, while PD-1/PD-L1 inhibitors often cause pneumonia and thyroid dysfunction. The most common adverse reactions of CTLA-4 inhibitors combined with PD-1/PD-L1 inhibitors were cutaneous adverse reactions and endocrine adverse reactions (43). Gastrointestinal complaints in the form of diarrhea and immune-mediated colitis are also common. Unusual common forms of acute kidney injury (AKI) include hepatotoxicity, endocrine disorders, and pneumonia, and rarer forms include nephrotoxicity, pancreatitis, neurotoxicity, cardiovascular toxicity, hematologic abnormalities, and ocular manifestations (28). Fatal irAEs are uncommon; a retrospective study of 3545 patients treated with ICIs at seven centers found mortality of only 0.6%, with cardiac and neurological events particularly prominent (43%). The fatal irAEs caused by anti-CTLA-4 were mainly diarrhea or enteritis (70%). The spectrum of fatal irAEs caused by anti-PD-1 and PD-L1 was broad, including pneumonitis (35%), hepatitis (22%), and neurologic toxicity (15%) (41)

Although the overall mean incidence of adverse event was similar for different cancer types, it also varied among drugs acting on different targets (42, 43). CTLA-4 inhibitors often cause colitis, hypophysitis and rash, while PD-1/PD-L1 inhibitors often cause pneumonia and thyroid dysfunction. The most common adverse reactions of CTLA-4 inhibitors combined with PD-1/PD-L1 inhibitors were cutaneous adverse reactions and endocrine adverse reactions (43). In addition, even drugs that act on the same target have different occurrences of IRAE; The same ICI produces different toxicity profiles when applied to different tumors. For example, nivolumab is easy to cause endocrine adverse reactions; arthritis, pneumonia and liver adverse reactions are common in pembrolizumab therapy (44), domestic camrelizumab is easy to cause reactive skin capillarosis, and PD-L1 inhibitor atezolizumab is more likely to cause hypothyroidism, nausea, vomiting and other symptoms (38). Studies have reported that melanoma patients have a higher risk of vitiligo than normal people, and the incidence is much higher than that of other tumor types. The general and specific irAEs of ICIs with different targets are summarized in **Table 2** (45–53).

**Table 2:** The general and specific irAEs of ICIs with different targets.

Targets	General	Distinct
CTLA-4	Colitis, pituitary inflammation and rash are commonly caused. Neurotoxicity (meningitis), hepatotoxicity, cardiotoxicity, hematotoxicity, and ocular toxicity are rare.	HLH is a fatal systemic inflammatory syndrome reported as a rare irAE in patients receiving nivolumab, ipilimumab, and/or pembrolizumab. Neuromuscular junction dysfunction (myasthenia gravis) was over-reported in patients treated with anti-PD-1/PD-L1 compared with anti-CTLA-4. Currently, 5 cases of acquired hemophilia A related to ICIs have been reported, including: ipilimumab, nivolumab, and atezolizumab. Camrelizumab: RCCEP, mainly manifesting as facial telangiectasia and the appearance of red blood streaks. Pembrolizumab: autoimmune polyendocrine syndrome.
PD-1/PD -L1	Cutaneous toxicity is the most common, followed by immune pneumonia, hypothyroidism, joint and muscle pain. PD-L1 inhibitor has a higher overall incidence of colitis. Myocarditis, immune nephritis and pituitary inflammation are rare yet serious.	
LAG-3	The main ones are colitis, immune hepatitis, rash, neuropathy, and endocrine toxicity.	

RCCEP, reactive cutaneous capillary endothelial proliferation; HLH, Hemophagocytic lymphohistiocytosis.

## 4 Mechanisms of irAEs associated with ICIs

It is apparent that irAEs share several similarities with autoimmune diseases. In line with this, a multitude of clinical case reports have demonstrated that ICIs can induce substantial autoimmune responses, akin to those manifested in autoimmune diseases. This implies that irAEs might represent subclinical autoimmune reactions in a subset of patients. The exact pathophysiological mechanism of irAEs remains unclear. At present, irAEs are believed to be related to changes in the function of the body’s autoimmune system, mainly including the breaking of autoimmune tolerance or the body becoming more sensitive to antigen recognition and attacking its own tissues (54). Multiple mechanisms have been proposed to explain the occurrence of irAEs, such as the production of autoantibodies, T-cell infiltration, and the mediation of inflammatory cytokines such as IL.

### 4.1 Autoreactive T cells

The balance between immune activation and immune tolerance maintains the normal function of immune regulation in the body, which is achieved through the costimulatory pathway of reactive T cells. Immune tolerance can suppress the activation of self-reactive T cells, playing a role in regulating the strength of the immune system. Inhibitory costimulatory molecules on naive T cells can regulate the balance between T-cell activation, tolerance, and immune-mediated tissue damage by binding to their ligand. ICIs can promote the activation and proliferation of T cells and eliminate the function of Treg cells, which play a crucial role in maintaining immune tolerance, the number of which is negatively correlated with the occurrence of irAEs (4, 55). ICIs inhibit immune checkpoint molecules to prevent immune escape of tumor cells and disrupt peripheral T-cell tolerance through the same mechanism, resulting in rapid diversification and clonal expansion of toxic cells (42) and high inflammation and autoimmunity (32, 56). Therefore, organs that rely heavily on peripheral T-cell tolerance to maintain immune homeostasis are the most common sites for irAEs, such as the skin and colon. Recently, a team led by Aaron M. Newman from Stanford University and Aadel A. Chaudhuri from the University of Washington School of Medicine discovered a correlation between high levels of CD4 effector memory T (TEM) cells in the blood and the development of severe irAEs. Their research suggests that activated CD4 TEM cells may be the basis of severe ICI toxicity. Furthermore, researchers found that in patients who experienced severe irAEs, the TCR clonal diversity in activated CD4 TEM cells was significantly increased, while this correlation was weak or absent in other T-cell subpopulations (57). It is also related to the type of ICI, and both CTLA-4 and PD-1 inhibitors increase the activation and proliferation of T cells and eliminate the function of Treg cells, which play a crucial role in maintaining immune tolerance. There is a negative correlation between the number of Treg cells and the occurrence of irAEs. Normal tissues that share antigens with tumor tissues are also susceptible to T-cell attack. Inhibition of CTLA-4 results in increased priming and activation of antigen-specific T cells, which can attack both malignant and nonmalignant tissues (58). Patients who responded well to ICIs had a higher proportion of CD45RO^+^CD8^+^ memory T lymphocytes and regulatory T lymphocytes. An increase in the proportion of CD4^+^ and CD8^+^ T lymphocytes during treatment was also associated with a good prognosis, possibly because the proportion of these T lymphocytes in the blood affects the antitumor immune response (59).

### 4.2 Autoreactive B cells

Activation of self-reactive B cells and production of self-antibodies increase, which may be newly generated or derived from pre-existing self-antibodies. These antibodies can bind to target antigens and cause damage, such as triggering the classical complement cascade reaction. The involvement of B lymphocytes was supported by another study that found 19.2% of patients who were negative for multiple antibodies before ICI treatment developed autoimmune antibodies after treatment. TPOAb and anti-thyroglobulin antibodies (TGAb) were the most common (60).

### 4.3 Cytokines

With the comprehensive development of tumor immunology, cytokines have entered a new era of development. Multiple cytokines, such as interleukins, tumor necrosis factor, and interferons, have become an essential part of tumor immunology. In patients with irAEs, certain cytokines undergo significant changes before and after treatment, which may be signal molecules partially enhanced by the immune system and play a role in patients with immune-related adverse events. The release of inflammatory mediators by immune cells can lead to immune-mediated damage in tissues with anatomical susceptibility, suggesting that tissue-specific or general cytokine levels may play a role in the pathogenesis of irAEs (61). These cytokines can bind to immune cells and activate intracellular signaling pathways (such as the JAK-STAT and PI3K-AKT-mTOR pathways), leading to dysregulated proinflammatory responses. There is also evidence that lower baseline IL-6 levels are significantly associated with the development of irAEs (62). In addition, the improvement of irAEs by TNF inhibitors also suggests that the mechanism is related to inflammatory factors (63). The microbiota plays an important role in irAEs by enabling the production of proinflammatory or anti-inflammatory cytokines, which are exacerbated after ICI treatment (54). Immune checkpoint inhibition to release symbiont-specific inflammatory T-cell responses was demonstrated by establishing a mouse model of commensal bacteria-driven cutaneous irAEs. These aberrant responses are dependent on symbiont-specific T cells to produce IL-17 and induce pathology recapitulation of the skin inflammation seen in patients treated with ICIs. Importantly, the aberrant T-cell responses released by ICIs are sufficient to perpetuate the inflammatory memory response to the microbiota several months after treatment cessation (64).

### 4.4 Host-specific factors

Finally, it is necessary to consider the impact of environmental factors, such as intestinal microbiota imbalance and the production of microbiota metabolites, which may cause abnormal activation of the immune system and easily lead to immune-related adverse events in ICI therapy.

**Figure 2:**
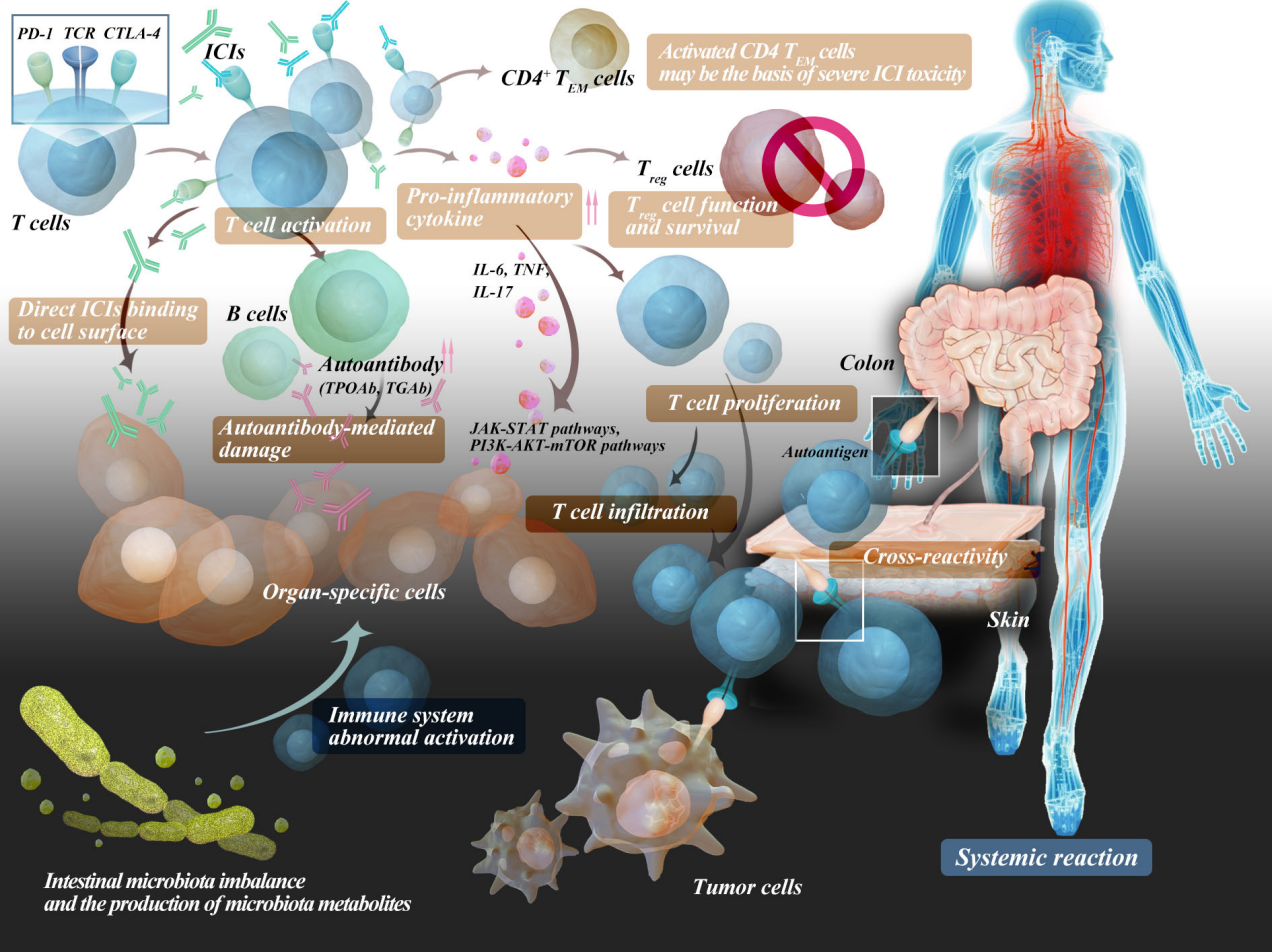
The potential adverse effect mechanisms of immune checkpoint inhibitors.

The possible mechanisms of adverse effects are shown in **Figure 2**.

## 5 Common irAEs

IrAEs involve various organ systems of the whole body, mainly including the skin system, digestive system, endocrine system, and respiratory system. Rare cases include nervous system toxicity and cardiac toxicity.

### 5.1 Cutaneous irAEs

Cutaneous immune-related adverse events (irCAEs) are the most common and usually occur first. Maculopapular rash (MPR), pruritus, and lichenoid dermatitis are the most common types (65). The more common of these are eczematous, morbilliform, and lichenoid dermatoses, as well as vitiligo and pruritus. Less common adverse events included psoriasiform skin disorders, bullous disorders, and severe cutaneous adverse effects, including Stevens−Johnson syndrome, toxic epidermal necrolysis, drug reactions with eosinophilia, and constitutional symptoms. Due to the immune mechanism of ICIs, there are a variety of rheumatic adverse effects with cutaneous manifestations, such as scleroderma, dermatomyositis, cutaneous lupus erythematosus, and various vasculitides (66, 67). The incidence of cutaneous adverse effects was higher with CTLA-4 antibody (34%–42%) than with PD-1 antibody (44%–59%) with monotherapy but highest for combination therapy (59%–72%) (68, 69). Rash and pruritus were more common with anti-CTLA-4, whereas vitiligo was more common with anti-PD-1. The increased incidence of irAEs is more common in some patients with preexisting cutaneous autoimmunity (e.g., bullous pemphigoid, psoriasis, lupus). Maculopapular rash occurs in up to 60% of patients treated with CTLA-4 inhibitors, occurs in 24% after anti-PD-1 treatment, and may be a precursor to other cutaneous adverse effects (70). The skin presented with varying degrees of pruritus, erythematous spots, and dome-shaped papules, some of which coalesced into patches and plaques. The rash is usually present on the trunk and/or limbs, usually on the extensor surface. Bending of the skin, scalp, palms, and face is rarely involved (71).

### 5.2 Digestive irAEs

The pathological manifestations of gastrointestinal and hepatobiliary injuries caused by ICI treatment are extensive (72). Lower gastrointestinal side effects were more common than upper gastrointestinal side effects (73). The main gastrointestinal adverse reactions were diarrhea and enteritis, and enteritis was the most common. It may occur weeks or months after ICI therapy (74). Accompanying symptoms such as abdominal pain, fever, blood or mucus in the stool, nausea, and vomiting may also occur (75). The most common manifestations of irAEs involving the upper gastrointestinal tract are loss of appetite and nausea. Stomatitis, esophagitis, dysphagia, gastritis, vomiting, and gastroesophageal reflux disease may also occur in some cases. (76) The incidence of diarrhea ranged from 12.1% to 13.7%, and the incidence of colitis ranged from 0.7% to 1.6% in patients receiving PD-1 inhibitors. Gastrointestinal irAEs are more frequent and more severe in patients who receive CTLA-4 inhibitors than in those who receive PD-1 inhibitors, with rates of diarrhea ranging from 27% to 54% and colitis ranging from 8% to 22%. When these two inhibitors are used together, the incidence and severity of irAEs in the intestinal tract will be significantly increased (77, 78). In addition, the use of nonsteroidal anti-inflammatory drugs (NSAIDs) was associated with an increased risk of colitis associated with ICIs (79). The incidence of diarrhea and colitis increased with increasing ICI dose (80).

### 5.3 Immune-mediated hepatotoxicity

The mechanism of hepatotoxicity is currently unknown, and secondary activation of CD8+ cytotoxic T lymphocytes, various CD4+ T-cell populations, cytokines, and the innate immune system has been found to lead to liver injury (81). According to the serum aspartate aminotransferase (AST)/alanine aminotransferase (ALT) levels, liver injury can be divided into five grades: (1) Grade 1 liver damage: AST/ALT increased, less than 3 times the upper limit of the normal value: the level of total positive rates increased less than 1.5 times the upper limit of the normal value. (2) Grade 2 liver injury: elevated AST/ALT, 3-5 times the upper limit of normal value; elevated serum total bilirubin, 1.5–3 times the upper limit of normal value. (3) Grade 3 liver injury: high AST/ALT (695–20 times the upper limit of normal value) and high carnosine (3.10 times the upper limit of normal value). (4) Grade 4: the level of AST/ALT was high, exceeding 20 times the next level, and the level of AST was increased to 10 times the upper limit of the normal value. (5) Grade 5 liver injury: fatal liver injury (31). Histologically, ICIs can cause various forms of pathological damage to hepatocytes, including panlobular hepatitis, perivenular infiltrating endotheliitis or a cholestatic pattern with proliferative bile duct injury, and mixed portal inflammation with mild lobular necrotizing inflammation (82). Hepatotoxicity has been reported in 2% to 10% of patients receiving monotherapy with ipilimumab, nivolumab, and pembrolizumab. Combination therapy with ipilimumab and nivolumab resulted in a 25% to 30% incidence of full grade hepatitis and a grade 3 toxicity rate of approximately 15%. The onset of the disease mainly occurs within the first 6 to 12 weeks after the initiation of treatment (83). In the context of ICI therapy, hepatitis is usually asymptomatic and manifests as an increase in ALT and/or AST levels (84). Most of the patients had spontaneous remission after stopping ICI treatment, and a few patients developed liver failure. Patients with more severe disease presented with fever, jaundice, right abdominal pain, dark urine, and easy abrasion. As mentioned earlier, acute liver failure (encephalopathy and coagulopathy) is rare, especially as an initial presentation (85). Rates of IMH of any grade were lowest with PD-1 (0.7%–2.1%), moderate with PD-L1 and standard-dose CTLA-4 (0.9%–12%), and highest with CTLA-4/PD1 (13%) and high-dose CTLA-4 inhibitor therapy (16%). The overall incidence of grade 3 and 4 IMH ranged from 0.6% to 11%, with high-dose CTLA-4 inhibitors being more common. Of note, cases of fulminant liver failure due to IMH (0.1%–0.2%) are rare (86).

### 5.4 Endocrine toxicity

Organs most commonly affected by ICI-related endocrine toxicity include the thyroid gland (which typically manifests as hypothyroidism, often secondary to thyroiditis; Graves’ disease-like hyperthyroidism is very rare), pituitary gland (hypophysitis or hypopituitarism), and islet beta cells (presentation similar to type I diabetes) (87). A systematic review/meta-analysis of 38 randomized trials with a total of 7551 patients showed that the overall incidence of clinically significant endocrine disease in patients treated with checkpoint inhibitors was approximately 10% (40).

#### 5.4.1 Thyroid disorders

Most thyroid dysfunction occurs 1–2 months after starting ICI therapy. Thyroid irAEs can be divided into thyrotoxicosis and hypothyroidism. Thyroiditis can occur during treatment with any type of ICI. Cytotoxic memory CD4 T cells activated by anti-PD-1 antibody injection play a key role in the pathogenesis of destructive thyroiditis in humans. No study has yet analyzed the mechanism by which anti-CTLA-4 antibodies induce thyroid irAEs (88). In a retrospective cohort study of 1246 patients treated with ICIs, ICI-related thyroid irAEs occurred in 518 (42%) patients. Subclinical thyrotoxicosis (n=234) was the most common thyroid irAE, followed by overt thyrotoxicosis (n=154), subclinical hypothyroidism (n=61), and overt hypothyroidism (n=39) (89). Thyroid dysfunction is the most common endocrine irAE in non-small cell lung cancer (NSCLC) (90). Primary hypothyroidism occurs in 6%–9% of patients treated with anti-PD-1 and/or anti-PD-L1, 4%–9% of patients treated with anti-CTLA-4, and approximately 16% of patients treated with anti-PD-1 (L)-1 and anti-CTLA-4 (91).

#### 5.4.2 Hypophysitis

Hypophysitis is a rare but important irAE that is often associated with symptoms such as fatigue, nausea, vomiting, weakness, headache, and gonadotropin deficiency, including loss of libido or erectile dysfunction. The rates of hypophysitis were 3.2% with ipilimumab, 0.4% with nivolumab or pembrolizumab, <0.1% with atezolizumab, and 6.4% with both nivolumab and ipilimumab (40). Hypophysitis is the most common irAE in patients treated with anti-CTLA-4 antibodies (approximately 5% of patients) and is more common in patients treated with the combination of ipilimumab and nivolumab, although the mechanism is not fully understood. Although hypophysitis usually affects women, ICI-related hypophysitis appears to be more common in male patients. It tends to appear within the first 2–3 months of treatment and even within 19 months after treatment. Symptoms associated with hypophysitis include fatigue, muscle weakness, headache, anorexia, nausea, weight loss, vision changes, temperature intolerance, arthralgia, and altered mental status. Hyponatremia, low adrenocorticotropic hormone (ACTH), or low thyroid stimulating hormone (TSH) may be present (92).

#### 5.4.3 Diabetes mellitus

ICI-associated diabetes mellitus, relatively rare, has an estimated incidence of 3.5% (93) and can become extremely severe, leading to irreversible damage to beta cells and even death if not recognized and appropriately managed in time. The precise mechanism of ICI-related diabetes is unknown. Symptoms in patients with ICI-related diabetes are diverse, ranging from asymptomatic hyperglycemia, polyuria, and polydipsia to diabetic ketoacidosis (DKA). The majority of patients were hospitalized for DKA, suggesting that this side effect is life-threatening. However, the study suggests that progressive DM does not significantly affect the survival of patients (48). In a systematic review and meta-analysis, investigators found that many people develop type 1 diabetes within 3 months of first exposure to PD-1/PD-L1 inhibitors. Patients with antibodies associated with type 1 diabetes have a more rapid onset of disease and a higher incidence of ketoacidosis than those without antibodies (94). Patients who received anti-CTLA-4 therapy were significantly less likely to develop diabetes than those who received anti-PD-1 or anti-PD-L1 therapy (95).

### 5.5 Neurotoxicity

The incidence of neurological irAEs has been reported to be approximately 1% (96). Although rare, it can have a significant impact on the quality of life of patients, accounting for 11% of secondary fatal events among irAEs (41), and therefore deserves attention, mainly including neuromuscular disorders, aseptic meningitis/encephalitis, peripheral neuropathy, and ophthalmic lesions. A systematic review noted that combination therapy with PD-1 mAb and CTLA-4 mAb led to the highest incidence of NAEs, followed by anti-PD-1/PD-L1 treatment, and anti-CTLA-4 treatment led to the lowest incidence, with incidence rates of 12%, 6.1%, and 3.8%, respectively (97). Anti-PD-1/PD-L1 therapy mostly leads to myasthenic syndromes, meningitis and cranial neuropathy, and rarely encephalitis and myositis; anti-CTLA-4 treatment mostly causes meningitis and less commonly encephalitis and myositis (98).

### 5.6 Cardiotoxicity

As a new type of antitumor therapy, such as traditional chemotherapy, ICIs also have toxic effects on the cardiovascular system, including myocarditis, heart failure, heart block, myocardial fibrosis and cardiomyopathy. However, the cardiac toxicity of ICIs is not as impressive as that of traditional chemotherapy drugs, especially anthracyclines, and is largely underestimated (99). Furthermore, the evaluation of sensitive and specific markers of cardiotoxicity, troponin and troponin, is not routinely performed in most immunotherapy trials, leading to difficulties in judging the actual incidence of early or late cardiotoxicity associated with ICIs (100). Studies have shown that PD-1 and PD-L1 are expressed in rodent and human cardiomyocytes, and disruption of the PD-1 coding gene in mice leads to dilated cardiomyopathy. Deletion of CTLA-4 and PD-1 lead to autoimmune myocarditis (56, 101, 102). In two models of T-cell-dependent myocarditis, PD-1 protects against inflammation and cardiomyocyte damage (102). Blockade of PD-1/PD-L1 signaling is also associated with other forms of cardiac diseases and suspected to mediate myocardial inflammation after acute myocardial infarction, exacerbating the formation of atherosclerotic plaques and leading to an increase in cardiovascular adverse events in patients (103). Several cases of myocarditis and even fatal heart failure have been reported in patients treated with monotherapy or a combination of ICIs over the past few years (104, 105). Another possible reason is that activated T cells may produce excessive IFN-γ, granzyme B, and TNF-α, which may lead to heart damage and be aggravated by ICIs blocking the negative regulation of T cells (99). Therefore, blockade of TNF-α may serve as an approach to prevent the manifestation of ICI-related cardiotoxicity (106). Interestingly, to date, all reported cases of ICI-associated cardiotoxicity have occurred in the first year following ICI infusion, and it remains to be seen whether delayed chronic cardiotoxicity will occur (56, 107, 108).

### 5.7 Renal toxicity

Nephrotoxicity of ICIs is less common than toxicity involving the skin, gastrointestinal tract, and endocrine system but is often underestimated due to diagnostic difficulties. The PD-1/PD-L1 pathway plays a key role in preventing improper immunity in kidney tissue, which typically exhibits increased PD-L1 expression. There is growing evidence that excessive activation of PD-L1 prevents the development of autoimmune nephritis and glomerulonephritis (109, 110). AKI is usually caused by acute interstitial nephritis related to ICIs, occurs in a minority of patients and can affect one or more compartments of the kidney (glomeruli, proximal/distal tubules, and interstitial tissue) (111, 112). The CTCAE system identified five grades of AKI based on serum creatinine (sCr) levels (31). In a meta-analysis of 5722 patients, data showed a higher incidence of nephrotoxicity associated with PD-1 mAb and that patients with urothelial carcinoma treated with pembrolizumab were more likely to develop kidney damage (113). Although ICI-AKI appears to be infrequent, combination therapy (anti-CTLA-4 plus anti-PD-1/PD-L1 or ICI plus chemotherapy) increases risk (114). Other types of kidney damage, such as IgA nephropathy and renal tubular acidosis, may also be associated with ICIs. Electrolyte abnormalities, including hyponatremia, hypocalcemia, hypokalemia, and Fanconi syndrome, require vigilant monitoring to avoid life-threatening complications. Management of renal irAEs is based on steroid administration and/or interruption of ICIs to prevent irreversible organ damage (115).

### 5.8 Adverse reactions of the respiratory system

Respiratory toxicity due to ICIs is frequently reported. The incidence of respiratory irAEs correlates with specific tumor types, including an increased incidence observed in NSCLC patients, with 17% of NSCLC patients reporting having at least one respiratory irAE (116). To date, more than 36,000 ICI-related respiratory irAEs have been collected and recorded, of which 75.4% occurred in the first 3 months of ICI treatment (the median onset time was 36 days), with a higher incidence in men than in women (possibly because the incidence of lung cancer in men is higher than in women). Anti-PD-1 and anti-PD-L1 therapy were significantly associated with respiratory toxicity, while the causal relationship between anti-CTLA-4 drugs and respiratory toxicity was not significant. Interstitial lung diseases and pneumonia were significantly associated with all ICIs. In addition, 7 of the 10 specific respiratory AEs (lower respiratory tract disease, pleural disease, pulmonary vascular disease, unclassified respiratory disease (NEC), respiratory infection, respiratory tumor, and chest disease) are associated with ICIs (117).

## 6 Management of irAEs

With the promotion and application of ICIs, medical practitioners must improve their understanding and management of irAEs.

### 6.1 Early identification

The research and application of ICIs is becoming increasingly extensive, but only a small proportion of patients with a small number of tumor types respond to and benefit from ICI treatment, which causes unnecessary adverse reactions and wastes resources. Therefore, whether there is a way to distinguish between ICI beneficiaries and intolerance is a hot topic of discussion. The majority of irAEs are reversible if diagnosed in a timely manner (118). Some biomarkers can identify the beneficiary groups and predict the therapeutic effects and adverse reactions of ICI therapy, thus helping to take countermeasures in advance to reduce immune-related damage (119). If biomarkers related to irAEs can be used to predict and monitor potential risks for patients and effective prevention and intervention measures can be taken in a timely manner, the deterioration of adverse events can be avoided, thereby ensuring the safety of subjects and smoothly conducting clinical trials, which is of great significance. The increase in expression of CD177 and CEACAM1, for instance, is closely related to colitis after ipilimumab treatment (120). Three currently FDA-approved predictive biomarkers, PD-L1, microsatellite instability (MSI), and tumor mutational burden (TMB), are routinely used for patient selection for ICI response in clinical practice (13, 121). However, the variables involved in applying the mentioned biomarkers have posed serious challenges in daily practice. Further studies are needed for potential biomarkers useful to perform such prediction, such as mismatch repair (MMR) deficiency (122), interferon-γ (IFN-γ)-related mRNA profile (123), and T-cell invigoration to tumor burden ratio (124). Furthermore, four novel gene signature biomarkers, the T-cell inflamed gene expression profile (GEP), T-cell dysfunction and exclusion gene signature (TIDE), melanocytic plasticity signature (MPS), and B-cell focused gene signature, have been reviewed, and it has been concluded that MPS shows the best predictive performance, followed by GEP and TIDE, which are superior to PD-L1 and TMB (121).

### 6.2 Interventions

The management of irAEs follows an approach similar to that of autoimmune diseases and, more specifically, similar to treatment for autoimmune disease exacerbations. Steroids, immunomodulators, and immune-oncologic (IO) discontinuation are cornerstones of irAE management, while an array of immune-suppressing agents can be used. Several immunomodulating agents commonly used in rheumatism are available for specific and severe irAEs. The exact approach and dosing depend on the severity and subtype of irAE presented (39, 125).

Based on the recommendations of the ASCO guidelines (83), better management of irAEs begins with educating patients and caregivers before and during ICI therapy, including but not limited to the mechanism of ICIs and the symptomatic manifestations and basic management principles of potential irAEs. In general, with the exception of some neurological, hematological, and cardiac toxicities that should be immediately discontinued, grade I irAEs should be closely monitored during ICI treatment. If irAEs occur, discontinuation or permanent discontinuation of ICIs should be considered according to the level of toxicity, and dose adjustment is not recommended. Most grade II irAEs can be considered with the addition of corticosteroids (initial dose 0.5 to 1 mg/kg/day prednisone or equivalent); if interrupted, ICI therapy should be restarted when symptoms and/or laboratory data are restored ≤ level I (the same applies to other toxicity levels of discontinuation of ICI therapy). For grade III irAEs, if ICI therapy is not interrupted, high-dose corticosteroids (prednisone 1–2 mg/kg/day or equivalent corticosteroids) should be started and tapered over 4 to 6 weeks. If symptoms do not resolve within 2 to 3 days, immunosuppressants such as infliximab may be used. When symptoms and/or laboratory data of patients treated with combination therapy of ICIs return to ≤grade I, restimulation can be performed with PD-1/PD-L1 monotherapy. In general, grade IV toxicity should be permanently discontinued with the exception of endocrine disorders that have been controlled by hormone replacement therapy. Under the current treatment guidelines, most immune-related adverse events (irAEs) can be controlled and reversed, and the treatment time is usually 4 to 8 weeks. However, some endocrine diseases are special situations that require long-term use of hormone replacement therapy. In addition, even in experienced medical centers that comply with toxicity management guidelines, serious adverse reactions may still occur. For example, despite the early use of corticosteroids and anti-TNF-a treatment, 1% of melanoma patients receiving ipilimumab still have intestinal perforation. The management of adverse effects of specific organs is summarized in **Table 3**.

**Table 3:** Management of irAEs in specific organs (74, 83, 126).

Targets	Actions
Cutaneous irAEs	
Maculopapule	I–II: ICIs continuation with topical intervention (emollient, antihistamine, intermediate-potency topical steroids) or oral prednisone 0.5 mg/kg/day II: highly effective topical steroids or oral prednisone 0.5 to 1 mg/kg/day III–IV: emergency dermatological consultation, potent topical steroids, hospitalization for IV; immunosuppressive therapies (aprepitant, omalizumab) if symptoms are refractory to steroids
BD, SJS, TEN	Dermatological consultation, skin biopsy I: discontinuation of ICI, highly effective topical steroids II: oral steroids 0.5–1 mg/kg/day (prednisone/methylprednisolone); rituximab is recommended if there is no improvement after 3 days III–IV: hospitalization; oral steroids 1–2 mg/kg/day (prednisone/methylprednisolone); IVIG 1/g/kg/day is recommended if intolerant to steroids
Gastrointestinal irAEs	
Intestines (Colitis/Diarrhea)	I: ICIs continuation; adequate hydration; loperamide or diphenoxylate/atropine for 2–3 days; evaluation of lactoferrin/calprotectin levels; mesalamine and cholestyramine in addition if no improvement II: oral steroids 1–2 mg/kg/day instead of GI motility agents; infliximab or vedolizumab is recommended if no response is observed in 2–3 days of steroid initiation III–IV: urgent GI consultation; hospitalization; IV methylprednisolone 1–2 mg/kg/day immediately; infliximab or vedolizumab in addition if no improvement
Liver	Grading is based on the degree of LFTs and bilirubin abnormalities relative to ULN 1–2 times ULN: ICIs continuation; oral steroids 1–2 mg/kg/day; mycophenolate in addition if no improvement after steroids 3–4 times ULN: inpatient care; urgent hepatology consultation; permanent ICI discontinuation; steroids administration (ditto)
Pancreas	asymptomatic patients: ICI continuation after active pancreatitis evaluation pancreatitis: ICI continuation, IV hydration and GI consultation for I–II; ICI discontinuation and oral steroids 0.5–1 mg/kg/day for III; permanent ICI discontinuation and oral prednisone/methylprednisolone 1–2 mg/kg/day for IV
Endocrine toxicity	
Hyperglycemia	Assess for new-onset hyperglycemia and, if so, DKA evaluation. If DKA-negative, continue ICI and control blood glucose through lifestyle; If DKA-positive, standard inpatient DKA management is recommended.
Thyroid	asymptomatic/subclinical hypothyroid states: ICI can be continued if TSH is 4–10 or elevated (>10) and T4 is normal. Levothyroxine is considered if TSH is >10 symptomatic thyrotoxic states: 10–20 mg q4–6 h or atenolol/metoprolol with repeat TFTs in 4–6 weeks; Graves’ disease evaluation
Hypophysitis	hormone replacement therapy; high-dose steroids is considered if any acute and severely symptoms
Neurologic irAEs	
Aseptic Meningitis/Encephalitis	ICI continuation for mild irAEs possible empiric treatment with IV acyclovir; prednisone 0.5–1 mg/kg/day (mild) or methylprednisolone 1–2 mg/kg/day (moderate-severe); rituximab in addition if no improvement is observed within 7–14 days of steroids
Peripheral Neuropathy	I: ICI continuation; symptom monitoring for a week II: close monitoring; 0.5–1 mg/kg/day steroids (–4 mg/kg/day methylprednisolone if any progression) Gabapentin, pregabalin, or duloxetine is recommended if any associated neuropathic pain
Cardiac irAEs	
	Immediate cardiac tests of ECG, echocardiogram, myocardial injury/inflammatory markers; Deactivation of ICI High-dose IV corticosteroids of 1 g/day (methylprednisolone) for 3 to 5 days, followed by oral corticosteroids for 4 to 6 weeks; immunomodulators (abatacept, mycophenolate, IVIG, alemtuzumab, infliximab, and anti-thymocyte globulin) in addition if steroids-refractory within the first 24 hours ICU admission and temporary or permanent cardiac pacing may be required at any level
Renal irAEs	
Nephritis	A significant increase in sCr levels during ICI treatment should be considered an indication of immune-associated nephritis, unless otherwise demonstrated. high doses of prednisone (> 1 mg/kg) for no more than 3 weeks or until baseline renal function is restored, and gradually reduce the dose within 5–6 weeks; immunosuppressant (azathioprine, cyclophosphamide, cyclosporine, infliximab, or mycophenolate) is recommended if no improvement
Pulmonary irAEs	
Pneumonia	I: ICI continuation, reassess relevant examinations within 1–2 weeks II–IV: minimally invasive examination to exclude infection; CT within 3 to 4 weeks; steroids (prednisone or methylprednisolone, 1–2 mg/kg daily) every 3–7 days III–IV: permanent discontinuation of ICI, oral corticosteroids (methylprednisolone, 1–2 mg/kg/day, tapering over 6 weeks or more); IVIG, mycophenolate mofetil or infliximab 5 mg/kg IV if there no improvement within 2 days

BD, bullous dermatitis; DKA, diabetic ketoacidosis; ECG, electrocardiograph; FBG, fasting blood glucose; LFTs, liver function tests; IV, intravenous; sCr, serum creatinine; SJS, Stevens-Johnson syndrome; TEN, toxic epidermal necrolysis; TFT, thyroid function test; TSH, thyroid stimulating hormone; ULN, upper limit of normal.

### 6.3 Promising targets and drugs for irAEs

According to current guidelines, steroids are the cornerstone of irAEs therapy, and ICI should be discontinued when severe symptoms occur. In addition, the American Society of Clinical Oncology and the European League Against Rheumatism recommend TNF-α inhibitors infliximab, CD20 inhibitor rituximab, and interleukin-6 receptor inhibitor tocilizumab as the preferred biological agents for the treatment of severe steroid-refractory irAEs (83, 127).

Infliximab targets TNF-α, one of the cytokines play crucial roles in inflammation and immune response. By inhibiting TNFα, these antibodies can help reduce inflammation and alleviate irAE symptoms. At present, although in lack of high-level evidence supported by reliable clinical trials, guidelines recommend the application of infliximab for the treatment of steroid-refractory ICI-related myocarditis and emphasize the risk of heart failure associated with infliximab (128). Therefore, TNF-α antibodies should be used with caution in patients with ICI-induced myocarditis.

Rituximab has therapeutic effects on steroids- and immunoglobulin-refractory neuro-related adverse events induced by ICIs. The clinical symptoms and nerve conduction of patients with multiple neurological diseases (129, 130) were significantly improved, and another patient with ICI induced myasthenia gravis (131) also benefited after rituximab treatment. In addition to the benefits of irAEs in the nervous system, rituximab also has an inhibitory effect on the reactivation of primary membranous nephropathy (132) related to ICIs, ensuring stable renal function and sustained ICI anti-tumor efficacy. Besides in the treatment of ICI mediated irAEs, rituximab is often used in combination with PD-1 inhibitors for follicular lymphoma, and its safety and activity have been confirmed in multiple clinical trials (133–135).

IL6 is an inflammatory cytokine produced by various cells and participates in the pathogenesis of immune disorders, exerting multiple effects (136). It is believed that anti-IL6 therapy reduces inflammation and has anti-angiogenic effects, with therapeutic effects in Castleman’s disease and inflammatory diseases (rheumatoid arthritis) without significant toxicity (137). Tocilizumab is currently used for the treatment of rheumatoid arthritis (138) and giant cell arteritis (139). Clinical trials have found that it is beneficial for irAEs of respiratory (140) and digestive (141, 142) systems, and can effectively control severe irAEs (such as myocarditis and large vessel vasculitis). Recently, it has also been approved for the treatment of immune dysfunction related to chimeric antigen receptor T-cell therapies (143). Study has shown that the application of tocilizumab in steroid-refractory irAEs alleviates clinical symptoms and significantly reduces treatment costs compared to infliximab (140). Similar to the results of previous study, a multicenter case study in 2021 also showed that tocilizumab can benefit patients with different cancers from irAEs lowering the effectiveness of anti-tumor treatments (144). In addition, tocilizumab also has therapeutic effect on cancer-related cachexia, and may have a synergistic anticancer effect with ICI treatment (145).

Similarly, other anti-IL antibodies such as the IL-17 inhibitor bimekizumab and secukinumab, and the IL-23 inhibitor guselkumab, play important roles in inflammation and immune responses. By inhibiting different family members of IL, these antibodies potentially alleviate irAE symptoms. IL-17A and IL-17F are key cytokines that promote the inflammatory process, with similar pro-inflammatory functions, driving chronic inflammation and damage in multiple tissues. Bimekizumab (146–148) can effectively and selectively inhibit IL-17A and IL-17F, being currently in the phase III clinical trial stage for the treatment of various inflammatory diseases, including plaque psoriasis, psoriatic arthritis. However, weakened immune monitoring may have a negative impact on tumor therapy after immunosuppressive treatment of irAEs, as secukinumab inhibits the efficacy of pembrolizumab in colorectal cancer with immune related psoriasis (149).

CTLA-4 negatively regulates T cell activation in various ways (150), and regulating CTLA-4 function is a promising strategy for immunotherapy of autoimmune diseases such as rheumatoid arthritis. In clinical practice, CTLA-4 immunoglobulin (Ig) fusion protein has been applied for autoimmune therapy (151). Blocking CTLA-4 in mouse model of autoimmune encephalomyelitis (152) enhances T cell activation and accelerates epitope transmission, thereby exacerbating disease recurrence. Abatacept is a recombinant fusion protein that contains the extracellular domain of human CTLA-4 and the modified Fc region of human IgG1. *In vitro* and *in vivo* studies have shown that Abatacept inhibits T cell proliferation and activation, demonstrating safety and tolerance in the treatment of rheumatoid arthritis (153, 154) and multiple sclerosis (155) patients.

By regulating relevant targets, the above drugs can potentially alleviate symptoms of irAEs and provide better intervention measures for irAEs, and providing more insights into the future application prospects of ICIs (128). As mentioned earlier, although these novel drugs and targets have shown hope in small-scale studies and animal models, it should be noted that cytokine inhibitors affect multiple aspects of the immune system, including infection and anti-tumor immunity. Incorporating them into the treatment of irAEs has both advantages and disadvantages, and further researches are needed to determine their safety, efficacy, and applicability for larger patient populations (156). Additionally, the development and testing of new drugs and targets for managing irAEs is an ongoing process, and new discoveries may emerge as research progresses.

## 7 Summary

The discovery of immune checkpoint pathways and the subsequent development of corresponding inhibitors over the past decade have been revolutionary breakthroughs in the field of cancer treatment. ICIs have ushered in a new era of antitumor therapy and greatly improved the survival rate of cancer patients. Nevertheless, over half of the patients do not benefit from ICI treatments, and they often come with immune-related adverse events (irAEs), which refer to toxicities caused by the immune system. Cutaneous irAEs are the most common, followed by endocrine irAEs. It is worth noting that although the incidence of irAEs in the heart and nervous system is not high, the consequences are serious and therefore need to be taken seriously. If we can predict and monitor the risk of irAEs in patients through related biomarkers and take timely and effective preventive and intervention measures to prevent the deterioration of adverse events, it is of great significance to ensure the safety of the subjects and the smooth conduct of clinical trials. The current treatment for irAEs primarily involves the use of corticosteroids, immunosuppressants, and cytokine antagonists. However, these therapies may induce immune system suppression in patients, thereby attenuating their antitumor immune responses. At present, there remain many problems to be solved regarding irAEs, such as unclear mechanisms and biomarkers, how to identify irAEs earlier, and how to develop more refined individualized drug treatments for irAEs. There are many limitations of this article; it only introduces the main immune-related adverse reactions and summarizes the interventions, which need to be further studied and summarized. We require a more comprehensive understanding of the mechanisms and incidence of irAEs associated with ICIs, as well as the development of more effective and safer therapeutic strategies. This necessitates further clinical research and practice, as well as enhanced communication and education between physicians and patients, to better manage these adverse events and improve patients’ survival rates and quality of life. It is believed that with the wider application of immunotherapy and more in-depth research on immune checkpoint inhibitors and their related immune adverse reactions, the above problems can be solved to help clinicians better screen the patients who can best benefit from and make better use of immunotherapy.

## Author contributions

QY and LW designed this review. QY and LW collected the references and completed the manuscript. QY and LW drew the figures and tables. LB and YB revised our initial manuscript and provided valuable comments. All authors contributed to the article and approved the submitted version.

## Glossary

**Table:**

ACTH	adrenocorticotropic hormone
AKI	acute kidney injury
ALT	alanine aminotransferase
APCs	antigen presenting cells
AST	aspartate aminotransferase
BCC	basal cell carcinoma
BD	Bullous dermatitis
BRCA	bladder urothelial carcinoma
CC	cervical cancer
cHL	classical Hodgkin’s lymphoma
CRC	colorectal cancer
CSCC	cutaneous squamous cell carcinoma
CTCAE	the Common Terminology Criteria for Adverse Events
CTLA-4	cytotoxic T lymphocyte-associated antigen 4
DKA	diabetic ketoacidosis
dMMR	mismatch repair deficiency
EC	endometrial carcinoma
ECG	electrocardiograph
EMA	European Medicines Agency
ESCC	esophageal squamous cell carcinoma
FDA	the United States Food and Drug Administration
FBG	fasting blood-glucose
GC	gastric cancer
GEP	gene expression profile
HC	Health Canada
HCC	hepatocellular carcinoma
HL	Hodgkin’s lymphoma
HLH	Hemophagocytic lymphohistiocytosis
HNSCC	head and neck squamous cell carcinoma
ICIs	immune checkpoint inhibitors
IMH	immune-mediated hepatotoxicity
irCAEs	cutaneous immune-related adverse events
irAEs	immune-related adverse events
IO	immune-oncologic
IV	intravenous
LAG-3	lymphocyte-activation gene 3
LFTs	liver function-test
MCC	Merkel cell carcinoma
MHLW	Ministry of Health, Labour and Welfare of Japan
MSI-H	high microsatellite instability
MPM	malignant pleural mesothelioma
MPS	melanocytic plasticity signature
MPR	Maculopapular rash
NK	natural killer cells
NMPA	the National Medical Products Administration of China
NPC	nasopharyngeal carcinoma
NSCLC	non-small cell lung cancer
PD-1	programmed death 1
PD-L1	programmed death-ligand 1
PMBCL	primary mediastinal large B cell lymphoma
PMF	primary myelofibrosis
RCC	renal cell carcinoma
RCCEP	reactive cutaneous capillary endothelial proliferation
SCLC	small cell lung cancer
sCr	serum creatinine
SJS	Stevens-Johnson syndrome
TCR	T cell receptor
TEN	toxic epidermal necrolysis
TFT	thyroid function test
TIDE	T-cell dysfunction and exclusion gene signature
TIGIT	T cell immunoglobulin and ITIM domain protein
TIM-3	T cell immunoglobulin and mucin domain-containing protein 3
TMB-H	high tumor mutation burden
TNBC	triple-negative breast cancer
TNF-α	tumor necrosis factor-alpha
Treg	regulatory T cells
TSH	thyroid stimulating hormone
UC	urothelial carcinoma
UCEC	uterine corpus endometrial carcinoma
ULN	upper limit of normal
VISTA	V-domain Ig suppressor of T-cell activation;
